# Targeting trisomic treatments: optimizing Dyrk1a inhibition to improve Down syndrome deficits

**DOI:** 10.1002/mgg3.334

**Published:** 2017-09-20

**Authors:** Megan Stringer, Charles R. Goodlett, Randall J. Roper

**Affiliations:** ^1^ Department of Psychology IUPUI 402 North Blackford Street, LD 124 Indianapolis Indiana 46202‐3275; ^2^ Department of Biology IUPUI 723 West Michigan Street SL 306 Indianapolis Indiana 46202‐3275

**Keywords:** Down syndrome, DYRK1A, EGCG, genotype‐phenotype correlation, learning and memory, Trisomy 21

## Abstract

Overexpression of *Dual‐specificity tyrosine‐phosphorylated regulated kinase 1A* (*DYRK1A*), located on human chromosome 21, may alter molecular processes linked to developmental deficits in Down syndrome (DS). Trisomic *DYRK1A* is a rational therapeutic target, and although reductions in *Dyrk1a* genetic dosage have shown improvements in trisomic mouse models, attempts to reduce Dyrk1a activity by pharmacological mechanisms and correct these DS‐associated phenotypes have been largely unsuccessful. Epigallocatechin‐3‐gallate (EGCG) inhibits DYRK1A activity in vitro and this action has been postulated to account for improvement of some DS‐associated phenotypes that have been reported in preclinical studies and clinical trials. However, the beneficial effects of EGCG are inconsistent and there is no direct evidence that any observed improvement actually occurs through Dyrk1a inhibition. Inconclusive outcomes likely reflect a lack of knowledge about the tissue‐specific patterns of spatial and temporal overexpression and elevated activity of Dyrk1a that may contribute to emerging DS traits during development. Emerging evidence indicates that Dyrk1a expression varies over the life span in DS mouse models, yet preclinical therapeutic treatments targeting Dyrk1a have largely not considered these developmental changes. Therapies intended to improve DS phenotypes through normalizing trisomic *Dyrk1a* need to optimize the timing and dose of treatment to match the spatiotemporal patterning of excessive Dyrk1a activity in relevant tissues. This will require more precise identification of developmental periods of vulnerability to enduring adverse effects of elevated Dyrk1a, representing the concurrence of increased Dyrk1a expression together with hypothesized tissue‐specific‐sensitive periods when Dyrk1a regulates cellular processes that shape the long‐term functional properties of the tissue. Future efforts targeting inhibition of trisomic *Dyrk1a* should identify these putative spatiotemporally specific developmental sensitive periods and determine whether normalizing Dyrk1a activity then can lead to improved outcomes in DS phenotypes.

## Introduction

Trisomy of human chromosome 21 (Hsa21) results in myriad phenotypes including cognitive impairment, cardiac abnormalities, and craniofacial features collectively referred to as Down syndrome (DS) (OMIM: 190685). Worldwide, DS affects 1 in 700–1000 live births (Parker et al. 2010). In nearly all cases of DS, three copies of ~300 genes found on Hsa21 occur in every cell beginning at conception and affect developmental processes in every system of the body. Phenotypes related to Trisomy 21 (Ts21) are apparent before birth and continue into old age. How three copies of genes on Hsa21 cause the phenotypes of DS is largely unknown. Although it has been assumed that trisomic genes are expressed at the dosage corresponding to chromosomal material in the cell (1.5 times that of normal, disomic genes) (Potier et al. 2006; Liu et al. 2008), multiple genetic mechanisms have been suggested for how the dosage imbalance of trisomic genes cause DS phenotypes (Potier et al. 2006; Roper and Reeves 2006; Antonarakis 2017). Hypothesized alternative mechanisms include suggestions that some trisomic genes or regions may be dosage sensitive and have a large effect on a particular DS phenotype, that trisomy globally alters gene expression throughout the genome, and that trisomy affects chromatin function (Roper and Reeves 2006; Korbel et al. 2009; Lyle et al. 2009; Ahmed et al. 2012; Letourneau et al. 2014; Antonarakis 2017). Owing largely to advances and applications in mouse models, the administration of therapies targeting phenotypes associated with DS has grown substantially over the past 15 years and have targeted diverse mechanisms (Stagni et al. 2015). To date, there have been over 20 different potential treatments administered to DS model mice in preclinical studies evaluating cognitive and behavioral phenotypes, with many treatments reporting improved phenotypes or symptoms observed in DS via targeting either specific neurotransmitter systems or aberrant neural pathways [for extensive review see (Gardiner 2015)].

Although a single trisomic genetic region or gene is not responsible for all DS phenotypes, there may be genes that have a major influence on phenotype(s) associated with DS due to the overexpression of a trisomic gene (Olson et al. 2004; Liu et al. 2008; Korbel et al. 2009; Lyle et al. 2009). One such gene, *Dual‐specificity tyrosine‐phosphorylated regulated kinase 1A*, is located on Hsa21 (*DYRK1A)* (OMIM: 60085), and mouse chromosome (Mmu)16 (*Dyrk1a),* and its overexpression has been linked to brain pathology in humans with DS and DS animal models (Dowjat et al. 2007; Liu et al. 2008). Consequently, *Dyrk1a* has been identified as a target for therapeutic drug development in DS (de la Torre and Dierssen 2012; Duchon and Herault 2016). Less consideration has been given, however, to when and where the trisomic gene is overexpressed, and whether that spatiotemporal regulation is causally related to the developing phenotype. Furthermore, many studies assessing therapeutics of Dyrk1a inhibition do not take into consideration the spatiotemporal regulation of the expression of Dyrk1a when administering Dyrk1a inhibitors to mouse models in DS. Consequently, few studies that administer Dyrk1a inhibitors (such as Epigallocatechin‐3‐gallate [EGCG]) directly examine concurrent Dyrk1a expression levels or the correlation between reduced Dyrk1a activity and therapeutic efficacy. This review examines the possible contributions of trisomic *Dyrk1a* to DS cognitive phenotypes, identifies major gaps in evidence needed to ascertain its putative role, and proposes a general strategy for developing rational treatments targeting trisomic genes to improve the developmental trajectory of DS.

## The Role of DS Mouse Models in Finding Therapies

Because of regions of homology between Hsa21 and Mmu16, Mmu17, and Mmu10 (Pletcher et al. 2001), various DS mouse models have been created [reviewed in (Das and Reeves 2011; Gupta et al. 2016; Xing et al. 2016)]. The use of mouse models with Hsa21 homologous genes in three copies has advanced efforts to correlate trisomic genes or regions with DS‐associated phenotypes. The Ts65Dn model consists of a segmental trisomy of Mmu16 that contains approximately 50% of the gene homologs found on Hsa21 (Davisson et al. 1990), and is the model used most often to test treatments to improve the various deficits observed in DS (Gardiner 2015). Successful outcomes from mouse models of DS have progressed to human clinical trials, due to the similarities in genetics (construct validity), particular phenotypes that are displayed (face validity) and new knowledge that may be applied to humans (predictive validity) (Rueda et al. 2012). However, results from large‐scale clinical settings have been generally disappointing. Lack of translational success from preclinical models to clinical trials is hardly unique to DS (Garner et al. 2017). Overall, there is a high failure rate (>80%) of clinical trials developed from preclinical findings in various mouse models of diseases (Gupta et al. 2016). Nevertheless, preclinical models of DS are likely essential for therapeutic advances, and it is critical to assess the rationale and mechanisms of targeted therapeutics in these mouse models because they provide the scientific and empirical foundation for clinical trials.

Many DS‐related approaches have largely focused on improving the phenotypes observed in DS. An alternative approach is to understand the influence of a trisomic gene product suspected to have a significant causative effect on the development of a phenotype and its aberrant mechanism, and develop treatments targeting those mechanisms (Ahmed et al. 2012). Therapeutics based on normalization of a single gene on an otherwise trisomic background to correct DS phenotypes is a significant paradigm shift, and has been supported by evidence from trisomic mouse models in which the normalization of one or two trisomic genes on an otherwise trisomic background from conception corrected some DS phenotypes (Cataldo et al. 2003; Hill et al. 2009; Chakrabarti et al. 2010; Blazek et al. 2015; Jiang et al. 2015; McElyea et al. 2016a; Kleschevnikov et al. 2017). The role of trisomic *Dyrk1a* in pathology associated with DS has been supported by the numerous reports of deleterious phenotypes that occur due to both over and underexpression of Dyrk1a in transgenic *Dyrk1a* mouse models (Arque et al. 2013) (see Table S1). Furthermore, *Dyrk1a* appears to have a crucial role during central nervous system development (CNS), via its regulation of multiple targets in both the nucleus and the cytoplasm via phosphorylation [extensively reviewed in (Becker et al. 2014; Duchon and Herault 2016)]. Normalization of *Dyrk1a* copy number in otherwise trisomic mouse models has resulted in some improvements in cognitive and behavioral phenotypes (Garcia‐Cerro et al. 2014).

Several recent reviews have discussed in detail the numerous targets of Dyrk1a*,* and have suggested how molecular mechanisms altered by excessive Dyrk1a could affect cognitive and behavioral processes (Wegiel et al. 2011; Park and Chung 2013; Duchon and Herault 2016; Antonarakis 2017). In addition, several reports have described potential Dyrk1a inhibitors and their possible use for correcting DS‐related deficits (de la Torre and Dierssen 2012; Becker et al. 2014; Duchon and Herault 2016). The present review extends those recent analyses by emphasizing an additional but crucial aspect of the therapeutic potential of Dyrk1a inhbition for DS – the importance of understanding when and where Dyrk1a expression and activity is elevated and determining whether some periods of elevated Dyrk1a may represent sensitive periods of developmental vulnerability for establishing long‐lasting DS structural and functional phenotypes. As a corollary, this review critically evaluates the literature on treatments using pure EGCG and EGCG‐containing supplements (the most frequently used putative Dyrk1a inhibitor to date) in DS mouse models, pointing to current limitations and additional information that is still needed to establish the full therapeutic potential of Dyrk1a inhibition.

## Function and Expression of Dyrk1a in Rodent Models

The expression of *Dyrk1a* mRNA and/or protein during normal (nontrisomic) development may provide insight into how overexpression of this kinase may lead to deleterious phenotypes. *Dyrk1a* is highly expressed during normal embryonic development, specifically in regions of the developing CNS (Martí et al. 2003; Hämmerle et al. 2008). The impact of *Dyrk1a* dosage alterations during development has been demonstrated in early studies reporting the embryonic lethality of knocking out *Dyrk1a* completely, with ~80% of homozygous *Dyrk1a* knockout mice (KO) dying *in utero* between Embryonic day (E)10.5–E13.5, and none surviving postnatally. Mice with only one copy of *Dyrk1a* present growth retardations and neurological deficits (Fotaki et al. 2002). Widespread expression of *Dyrk1a* protein was found in E17 mouse embryos and adult mice, but it was reported to be more abundant in regions of the CNS, specifically the cerebral cortex, cerebellum and hippocampus (Rahmani et al. 1998). Thus, while *Dyrk1a* protein may be ubiquitously expressed, there may be varying levels of protein within a specific tissue. This hypothesis is supported by the finding that levels of Dyrk1a protein expression in adult mice (aged 6 months–1 year) vary across different brain regions, with significantly higher Dyrk1a protein expression in the olfactory bulb and cerebellum compared to the cerebral cortex, hippocampus and hypothalamus (Martí et al. 2003). In addition, an extensive analysis of both *Dyrk1a* mRNA and protein expression during embryonic development revealed that expression of both mRNA and protein is dependent on spatial and temporal factors (Hämmerle et al. 2008).

While some studies report strong correlations between *Dyrk1a* mRNA and Dyrk1a protein levels across multiple tissues (Hämmerle et al. 2008), there can be mismatches in the level of gene expression and protein levels across time. For instance, during early postnatal development in Wistar rats (Postnatal day (P)1‐P21), *Dyrk1a* mRNA expression assessed via Northern blot was low in the cerebellum, whereas Dyrk1a protein expression assessed via Western blot was highest during these same time points. Later in development (P21‐adulthood), *Dyrk1a* mRNA expression in the cerebellum was high, and protein expression was reduced (Okui et al. 1999). Thus, although *Dyrk1a* mRNA expression may be prevalent throughout the brain, there can be significant differences in the regulation of gene expression and in the levels of protein, even within specific brain regions (Table S2). While there has not been a systemic analysis of both *Dyrk1a* mRNA and protein expression levels in the Ts65Dn mice, it is critical to note that mRNA/protein expression in trisomic humans and mice does not always follow the theoretical 1.5‐fold overexpression (Lockstone et al. 2007). Varying levels of Hsa21 gene dysregulation have been reported in both human and mouse tissue, and the degree of over or underexpression may depend on the age, as well as the type of tissue being sampled (Chrast et al. 2000; Bahn et al. 2002; Mao et al. 2003; Chou et al. 2008; Vilardell et al. 2011). For example, there were no reported differences in Dyrk1a mRNA levels between Ts65Dn and euploid mice at 5 months of age, but at 12 months of age Ts65Dn mice exhibited elevated levels compared to euploid animals (Choi et al. 2009). In addition, although Dyrk1a protein levels were significantly increased in three distinct brain regions in adult Ts65Dn mice, there was not the theoretical 50% increase in the hippocampus or cerebellum (31% and 24% increased, respectively) (Ahmed et al. 2012).

## Dissecting the Functional Role of *Dyrk1a* from Humans with Partial Trisomy and in Trisomic Mouse Models

Studies in humans and mice have attempted to correlate specific trisomic genes or regions and DS phenotypes. Although a single gene or genetic region is not responsible for all DS phenotypes (Lyle et al. 2004; Olson et al. 2004; Korbel et al. 2009), unraveling the contribution of a single trisomic gene such as *DYRK1A* has been complicated. In humans, trisomy of *DYRK1A* in isolation has not been reported, and therefore its singular effects on trisomic phenotypes may be underestimated (see the DYRK1A triplosensitivty score of 0 from the ClinGen Working Group Dosage Sensitivity Map [https://www.ncbi.nlm.nih.gov/projects/dbvar/clingen/clingen_gene.cgi?sym=DYRK1A&subject=]). Trisomy of only *DYRK1A* may be lacking due to limitations of recombination or detection in humans. Trisomic *DYRK1A* has been found in humans with partial trisomy of Hsa21 and trisomic regions including *DYRK1A* have been associated with DS phenotypes (Korbel et al. 2009; Lyle et al. 2009; Cetin et al. 2012; Papoulidis et al. 2014). From these data, it may be that *DYRK1A* may exert its deleterious effects in conjunction with other triplicated genes. Thus, inhibition of just *DYRK1A* may not completely improve a DS‐related phenotype. In addition, this could also suggest that the *DYRK1A* overexpression, and its deleterious effects may be specific to a defined window of development, and a specific tissue.

Transgenic *Dyrk1a* mouse models (on an otherwise euploid background) have provided information regarding the deleterious effects of overexpression of *Dyrk1a* itself (Table S1), but have some genetic caveats to understanding the function of Dyrk1a. It is important to note that there is variability in specific phenotypes of transgenic *Dyrk1a* mouse models, for example in motor development/performance. This could be due to differences in the behavioral tasks used to measure this phenotype, such as gait assessment, latency to begin walking, balance beam task, and various rotarod tasks. In addition, differences observed between transgenic *Dyrk1a* mice could be due to biological variations in the specificity of spatial expression of *Dyrk1a* between transgenic models, as different transgenic models express *Dyrk1a* under different promoters (Altafaj et al. 2001; Ahn et al. 2006). The age of assessment may also play a role in the differences between studies. For example, BACTgDyrk1a as compared to control mice exhibit decreased body weight and length on P30, but these same measures are not significantly different at P15 and P60 (Guedj et al. 2012). TgDyrk1a mice, one of the most utilized *Dyrk1a* transgenic models, appear to display age‐specific deficits on multiple developmental assessments, including walking, pivoting locomotion, and negative geotaxis (Arque et al. 2013). Interestingly, YACTgDyrk1a are not impaired on the Y‐maze task, which is thought to measure spatial working memory, whereas the BACTgDyrk1a mice are impaired, even though the mice were approximately the same age (~3 months) in comparative studies (Guedj et al. 2009; Souchet et al. 2014). The age and phenotype‐dependent differences evident in these mice suggest that the deleterious effects of Dyrk1a overexpression could depend on certain age(s), tissue(s), and means of phenotypic assessment.

Transgenic *Dyrk1a* mouse models are limited as models of DS because there are multiple genes that are dysregulated in DS, not just *Dyrk1a*. Consequently, a phenotype that is observed in *Dyrk1a* transgenic mice may have limited validity as a DS model and may not translate to a trisomic mouse model with many trisomic genes in three copies, due to the lack of interactions with other overexpressed or trisomic genes or differences in developmental trajectories between trisomic mice compared to *Dyrk1a* transgenic mice. In turn, other triplicated genes that are observed in trisomic models can make it difficult to understand the independent contribution of *Dyrk1a* overexpression to a specific phenotype in the Ts65Dn mouse. If the polygenic trisomic contributions are not understood, it would be difficult to develop a targeted therapeutic based on improvements in one or even multiple behavioral outcomes. While *Dyrk1a* transgenic mice highlight the importance of regulated *Dyrk1a* expression during development, studies of mouse models that are trisomic for *Dyrk1a* in conjunction with other Hsa21 homologous genes are crucial to better understand *Dyrk1a* expression and its association with DS traits.

Ts65Dn mice, with *Dyrk1a* and approximately 100 other genes in three copies, display some similar cognitive and behavioral deficits as transgenic *Dyrk1a* mice. With the triplication of many genes, it is important to determine the direct influence of *Dyrk1a* overexpression on Ts65Dn trisomic phenotypes. In contrast to mice only overexpressing *Dyrk1a*, or Ts65Dn mice that are trisomic for many genes, Ts65Dn mice crossed with *Dyrk1a* haploinsufficient mice, result in some Ts65Dn mice with a normalized copy number of *Dyrk1a* (Ts65Dn/Dyrk1a +/+/−) on an otherwise trisomic background (Garcia‐Cerro et al. 2014; Blazek et al. 2015; McElyea et al. 2016a). This gold standard methodology is used to understand the behavioral phenotypes that are improved in Ts65Dn/Dyrk1a +/+/− mice versus Ts65Dn/Dyrk1a +/+/+ (Ts65Dn) mice, which could be specifically attributed to the extra copy of *Dyrk1a* on a trisomic background (Table 1). Male Ts65Dn/Dyrk1a +/+/‐ mice (6–7 months old) exhibited a partial improvement in MWM latency, neuronal proliferation and differentiation, and hippocampal LTP. However, there were several phenotypes that were not rescued with normalization of *Dyrk1a* levels, including cell density of mature neurons in the dentate gyrus, dentate gyrus volume, or a variety of motor task deficits (Garcia‐Cerro et al. 2014). Subtracting one copy of *Dyrk1a* from another DS mouse model, Dp(16), (over half of the homologous genes on Hsa21 in three copies) showed that Dp(16) mice with one fewer copy of *Dyrk1a* performed better on T‐maze and contextual fear conditioning tests as compared to Dp(16) mice (Jiang et al. 2015). The lack of rescued motor task deficits is interesting, as administration of an adeno‐associated virus type 2 (AAVshDyrk1a) into the striatum lowering expression of *Dyrk1a* in TgDyrk1a mice normalized motor task deficits (Ortiz‐Abalia et al. 2008). A similar viral technique in 2‐month‐old Ts65Dn mice restored LTP deficits and normalized Dyrk1a protein levels in the hippocampus, but did not rescue MWM latency deficits (Altafaj et al. 2013). As with transgenic *Dyrk1a* animals, strain, methodological differences in the various tasks, or differences in the age of the subjects may account for outcome differences between studies (Table 1). Taken together, normalization of *Dyrk1a* does not appear to be responsible for all the deficient cognitive and behavioral phenotypes of trisomic mice. Thus, the influence of *Dyrk1a* on a specific phenotype in trisomic mice is complex, and other trisomic genes likely contribute to these phenotypes.

**Table 1:** The effects of normalizing *Dyrk1a* copy number in TgDyrk1a and Ts65Dn mice

Mouse model	Technique	Age	Area	Improved	Did not improve	Authors
Ts65Dn	shRNA	2 month	Hippocampus	LTP, initial thigmotaxic behavior	MWM latency, later thigmotaxic behavior	Altafaj et al. (2013)
TgDyrk1a	shRNA	2–3 month	Striatum	Hyperactive behavior, treadmill task, PPI	N/A	Ortiz‐Abalia et al. (2008)
Dp16	Crossed with Dyrk1a ^m1^/+ mice	2–4 month	Global	T‐maze task, contextual fear conditioning	N/A	Jiang et al. (2015)
Ts65Dn	Crossed with Dyrk1a +/− mice	5–6 month	Global	Some MWM latency, LTP, neuronal proliferation & differentiation	Fear conditioning, motor coordination, locomotor activity, open field anxiety, cell survival, DG volume, SGZ area, body weight	Garcia‐Cerro et al. (2014)

MWM, Morris water maze; LTP, long‐term potentiation; PPI, prepulse inhibition; DG, dentate gyrus; SGZ, subgranular zone; N/A, not available or done.

In addition, it is likely that *Dyrk1a*'s overexpression and subsequent influence could be strongest at a specific developmental time (for a given tissue) when developmental trajectories are established for the tissue. If so, then normalizing or reducing this overexpression at a specific time (for that tissue) may produce an optimal timing for a potential therapeutic. Although genetic reductions of *Dyrk1a* copy number from conception have shown significant corrections of DS phenotypes (Garcia‐Cerro et al. 2014; Blazek et al. 2015; Jiang et al. 2015; McElyea et al. 2016a), and provide proof‐of‐principle that *Dyrk1a* is a relevant target, pharmacological treatments targeting Dyrk1a activity to date have had only limited successes due to a limited understanding of the effects of spatial and temporal overexpression of trisomic *Dyrk1a*.

## Unresolved Questions about Dyrk1a

If a treatment is to target Dyrk1a overexpression for deficits observed in Ts21, several important questions need to be addressed. First, the levels of Dyrk1a protein and kinase activity in Ts65Dn mice throughout development need to be ascertained; these levels are not well known, especially during perinatal and young adolescent stages (Table 2). This emphasizes the need to identify and understand the temporal and spatial regulation of *Dyrk1a* expression in trisomic mice, especially considering suggestions of the prenatal or neonatal developmental age as an optimal target window for improving deficits in DS (Guedj et al. 2014; Stagni et al. 2015). A recent study showed that the magnitude of protein abnormalities of both Hsa21 and non‐Hsa21 proteins were exacerbated in 12‐month‐old Ts65Dn mice versus ~6‐month‐old Ts65Dn mice (Ahmed et al. 2017). Specifically, in the hippocampus of Ts65Dn mice, Dyrk1a protein levels were only 30% higher than controls at 6 months of age, but these levels were 100% higher in the 12‐month‐old Ts65Dn mice, versus euploid controls (Ahmed et al. 2017). Temporal regulation of Dyrk1a protein levels has also been reported in the Ts1Cje mouse model in the cerebellum at three adult ages. Dyrk1a protein levels significantly increased with age in adult Ts1Cje mice (between 4, 12, and 17 months of age) (Créau et al. 2016). In embryonic day (E) 13.5 Ts1Cje embryos, there was increased Dyrk1a protein expression in cortical neurons. However, in whole brains of E11.5 Ts1Cje embryos and in hippocampal neurons of P1 Ts65Dn mice, there were no differences in levels of Dyrk1a protein (Arron et al. 2006). In addition, Dyrk1a protein expression in Ts65Dn mice at ~68 days of age did not show differences in protein expression between euploid and trisomic mice in the hippocampus or cerebral cortex. However, trisomic mice at this same age exhibited an unexpected significant decrease in Dyrk1a protein levels in the cerebellum (Stringer et al. 2017). Finally, expression of Dyrk1a protein itself may not be sufficient to identify its role in development, since its kinase activity may be the most proximal measure of its regulation of cellular dynamics. Overall, the influence of *Dyrk1a* is likely to be dependent on both spatial and temporal factors.

**Table 2:** Dyrk1a protein and kinase activity levels in various brain regions in Ts65Dn mice

Age	Area	Effect	Authors
E11.5	Telencephalon	Increased Dyrk1a protein levels	Najas et al. (2015)
1.5 month	Cerebellum, hippocampus	No difference in Dyrk1a kinase‐related activity	Stringer et al. (2015)
~2 month	Cerebellum, cortex, hippocampus	No difference in Dyrk1a kinase‐related activity	Stringer et al. (2017)
~2 month	Cerebellum	Ts65Dn mice exhibit decreased Dyrk1a protein levels, no difference in cortex or hippocampus	Stringer et al. (2017)
3.5 month	Hippocampus	Increased Dyrk1a protein levels	Altafaj et al. (2013)
4.4–7.8 month	Cerebellum, cortex, hippocampus	Increased Dyrk1a protein levels	Ahmed et al. (2012)
5–6 month	Hippocampus	Increased Dyrk1a protein levels	Garcia‐Cerro et al. (2014)
~6 month	Hippocampus, cortex, cerebellum	Increased Dyrk1a protein levels	Ahmed et al. (2017)
7–8 month	Cortex, hippocampus	Increased Dyrk1a protein levels	Siddiqui et al. (2008)
7–8 month	Brain homogenate	Increased Dyrk1a protein levels	Dowjat et al. (2007)
12 month	Hippocampus, cortex, cerebellum	Increased Dyrk1a protein levels	Ahmed et al. (2017)
13–14 month	Brain homogenate	Increased Dyrk1a protein levels	Kida et al. (2013)
15 month	Brain homogenate	Increased Dyrk1a protein and activity	Liu et al. (2008)

A second factor that needs to be addressed is the tissue and cellular specificity of *Dyrk1a* expression levels in trisomic mice. This is crucial to link the tissue specificity and timeline of overexpression of *Dyrk1a* to a specific phenotype. Patterns of age‐dependent structural and histological phenotypes that vary with brain region are now recognized (see Table S3 [cerebellum] and Table S4 [hippocampal formation]). Yet, the lack of systematic quantitative data for Dyrk1a protein and/or mRNA levels in specific tissues makes it difficult to fully ascertain the role of Dyrk1a in the development of a specific phenotype. For example, Dyrk1a may have been overexpressed earlier in development that preceded or led to the phenotypic changes in the cerebellum evident at a later age. As another example, it is reasonable to hypothesize that MWM deficits reported in some studies of trisomic mice would be associated with a concomitant elevation in *Dyrk1a* in the hippocampus at the age of testing (Garcia‐Cerro et al. 2014; de la Torre et al. 2014; Stringer et al. 2015; Stringer et al. 2017). If so, this could directly implicate Dyrk1a overexpression in the hippocampus for MWM deficits and provide a means to test correlations between Dyrk1a activity and cognitive therapeutics with inhibitors. These examples highlight the need to identify whether overexpression of Dyrk1a at the time of behavioral testing is associated with a given phenotype, or identify whether a period of overexpression earlier in development (that subsequently resolved prior to testing) may be associated with the deficient phenotype. Because the influence of *Dyrk1a* overexpression on particular phenotypes appears to be dependent on age, it will be important to establish the developmental periods during which *Dyrk1a* is overexpressed and the specific brain systems in which it occurs, before causal links to the specific structural and behavioral phenotypes can be inferred. More detailed studies of these spatio‐temporal differences in Dyrk1a expression and activity are a prerequisite to identify an appropriate therapeutic window to target.

It will also be important to address how the expression of *Dyrk1a* during a developmental process directly results in a specific behavioral phenotype. For example, the relationship between Cyclin D1 and Dyrk1a is an attractive mechanism because Cyclin D1 and Dyrk1a protein levels have been reported in Ts65Dn mice at a specific age and region (Najas et al. 2015). Dyrk1a protein levels were significantly increased in E11.5 Ts65Dn mice, whereas Cyclin D1 protein levels were significantly decreased. Documentation of Dyrk1a protein levels in a specific tissue, combined with known levels of a potential target in Ts65Dn mice, is rare and could facilitate identification of the relationship between the level of Dyrk1a activity and a particular phenotype. For example, *Dyrk1a* directly regulates Cyclin D1, and normalization of *Dyrk1a* expression resulted in a rescue of both progenitor production and neuronal differentiation (Najas et al. 2015).

The direct link of *Dyrk1a* overexpression to a specific mechanism described above suggests that normalization of Dyrk1a could lead to a rescue of a specific phenotype. One hypothesis is that normalization of the embryonic and postnatal cell cycle would result in an improved cognitive‐based task. It remains unknown whether correction of a prenatal cellular phenotype, like embryonic progenitor production or neuronal maturation, will correct deficient postnatal phenotypes that subsequently emerge (like LTP or spatial memory). Furthermore, whether a prenatal treatment that corrects a developmental cellular or structural deficiency would also improve a postnatal behavioral phenotype is still unknown. Given the importance of Dyrk1a regulation of major neurodevelopmental processes, and the distinct likelihood that the postnatal trajectory of altered brain structural and functional development in DS has its origins in fetal or postnatal Dyrk1a overexpression, it is essential to identify the substrates or secondary targets of Dyrk1a regulation that are implicated in deleterious neurodevelopmental phenotypes. For example, studies that normalized *Dyrk1a* levels at various ages in different strains of mice demonstrated that the overexpression of this gene is implicated in motor, as well as cognitive‐based tasks such as the MWM (Ortiz‐Abalia et al. 2008; Altafaj et al. 2013; Garcia‐Cerro et al. 2014). However, with the heterogeneity in mouse strains, *Dyrk1a* normalization methodologies and the different ages of the animals, it is difficult to ascertain what specific phenotype(s) the *Dyrk1a* dosage imbalance is influencing. Najas et al. (2015) demonstrated that the normalization of *Dyrk1a* in Ts65Dn mice significantly improved embryonic neurogenesis deficits, and future studies should examine whether this improved neurogenesis also results in an improved behavioral phenotype.

## EGCG and Dyrk1a Inhibition

*DYRK1A* has become a target for DS drug development (Duchon and Herault 2016) and several molecules have been identified or developed to inhibit DYRK1A activity, including harmine, EGCG, INDY, FINDY, leucettine L41and CX‐4945 (Adayev et al. 2011; Ogawa et al. 2010; Kii et al. 2016; Kim et al., 2016; Fant et al. 2014; Naert et al. 2015). These molecules may have limited pharmacotherapeutic value due to side effects or off‐target effects [e.g., harmine has side effects associated with monoamine oxidase A (MOA) inhibition (Kim et al. 1997)]. Others have only recently been developed and have not undergone extensive preclinical testing.

One inhibitor of DYRK1A activity that has been extensively used in preclinical and in human clinical studies is EGCG. EGCG, the most common green tea polyphenol, inhibits DYRK1A activity in vitro (Bain et al. 2003). A relatively safe drug profile, combined with its ability to inhibit DYRK1A in vitro*,* and the translational value of comparative studies between animal models and clinical settings, support the current enthusiasm for pursing EGCG treatment to improve phenotypes observed in Ts21 (Bain et al. 2003; Adayev et al. 2006; Smith 2011). Although EGCG was originally administered in the DS setting for its ability to inhibit DYRK1A activity, the field has largely failed to determine whether EGCG treatment – usually administered as part of a supplement either to trisomic mice or to individuals with DS – actually improves the behavioral and cellular deficits via inhibition of Dyrk1a activity, the putative mechanism of its effects.

## Heterogeneity in Behavioral Outcomes after EGCG and EGCG‐containing Supplement Administration

Several studies that have administered EGCG or EGCG‐containing supplements to transgenic *Dyrk1a* or Ts65Dn mice have reported improved behavioral outcomes [Table 3, also see (Stagni et al. 2017)]. The amount of heterogeneity among studies across multiple variables is striking, involving different mouse models, ages, doses and composition of the EGCG treatment, as well as different behavioral tasks and outcomes. At a minimum, all studies should control or monitor the amount and composition of the EGCG treatment administered to the subjects and identify the effective dose and levels of EGCG achieved in the subjects over time. Many studies have used EGCG‐containing supplements as the source of EGCG, and there are several supplements that contain additional components such as other catechins, sucrose, and/or caffeine. The other catechins found in EGCG‐containing supplements, such as epigallocatechin (EGC), epicatechin gallate (ECG) epigallate (EG), and epicatechin (EC) could be acting synergistically with EGCG, or could be exerting effects independently of EGCG (Abeysekera et al. 2016). For example, EGCG‐containing supplements exhibit differential effects on various skeletal measures, with some supplements improving trabecular structure, yet others being detrimental to bone strength (Abeysekera et al. 2016). This demonstrates that the other components of EGCG‐containing supplements may contribute to the variable outcomes of treatment, depending on the specific phenotype that is assessed. Before mechanistic interpretations can be inferred, it is imperative that the independent effect of EGCG on Ts65Dn mice is evaluated.

**Table 3:** The effect of EGCG and EGCG‐containing supplements on Dyrk1a kinase activity and cognitive phenotypes in transgenic *Dyrk1a* and Ts65Dn mice

Treatment	Mouse model	Age	Dose	ROA	Length	Improved deficits	No effect	Tissues examined	Effect on Dyrk1a kinase levels	Authors
EGCG (>95% EGCG)	Ts65Dn	3 week	10, 20 mg/kg/day	DW	21D	N/A	MWM (Latency, path length, probe performance) Balance beam, locomotor activity, NOR	Cerebral cortex, hippocampus, cerebellum	None	Stringer et al. (2015)
EGCG (>95% EGCG)	Ts65Dn	3 week	50 mg/kg/day	DW	49D	N/A	MWM (Latency, path length, probe performance) Balance beam, NOR, MCSF	Cerebral cortex, hippocampus, cerebellum	None	Stringer et al. (2017)
EGCG (>95% EGCG)	Ts65Dn	P3	25 mg/kg/day	Inject	13D	Improved proliferation, connectivity in neocortex and hippocampus at P15	Improvements seen at P15 disappeared at P45 examination No improvements in Y‐maze or MWM at P45			Stagni et al. (2016)
Green tea polyphenols (Tea from Pacific Co. Ltd.)	YACTgDyrk1a	E0	0.6–1.0 mg/day	DW	90D	Improved NOR deficits	N/A	Hypothalamus‐thalamus	None	Guedj et al. (2009)
GTE (45% EGCG)	BACtgDyrk1a	12–16 week	120–200 mg/kg/day	DW	28–42D	Normalized LTP & spine density	N/A			Thomazeau et al. (2014)
GTE (45% EGCG)	Ts65Dn (Female)	20–24 week	30 mg/kg/day	DW	30D	N/A	MWM latency, increased thigmotactic behavior, reduced swimming speed			Catuara‐Solarz et al. (2015)
Decaffeinated MGTE (45%EGCG)	TgDyrk1a (Female)	3 week	2–3 mg/day	DW	30D	Hippocampal cell proliferation	N/A			Pons‐Espinal et al. (2013)
Decaffeinated MGTE (45% EGCG)	BACtgDyrk1a	12–16 week	60 mg/kg/day	Chow	28D	Spontaneous alternation	Exploratory activity in the Y‐maze			Souchet et al. (2015)
MGTE Lightly Caffeinated (45% EGCG)	Ts65Dn	3 week	2–3 mg/day	DW	30D	MWM (Latency & thigmotaxis) & NOR exploration	N/A			de la Torre et al. (2014)
MGTE Lightly Caffeinated (45% EGCG)	TgDyrk1a	12W	2–3 mg/day	DW	30D	MWM (Latency & thigmotaxis) & NOR exploration	N/A	Hippocampus	Decreased	de la Torre et al. (2014)
POL60 (27% EGCG)	YACTgDyrk1a	E0	1.2 mg/day	DW	90D	Improved NOR performance	N/A			Guedj et al. (2009)
POL60 (27% EGCG)	Ts65Dn	12–16 week	60 mg/kg/day	DW	28D	Spontaneous alteration	N/A			Souchet et al. (2015)

DW, drinking water; MWM, Morris water maze; NOR, novel object recognition; MCSF, multivariate concentric square field; LTP, long‐term potentiation; GTE, green tea extract; MGTE, mega green tea extract; N/A, not available or not done.

Transgenic *Dyrk1a* mouse models (TgDyrk1a, BACTgDyrk1a, YACTgDyrk1a) have been widely used to test EGCG or EGCG‐containing supplements, and all of these studies have reported improved behavioral outcomes. This is in stark contrast to our studies using Ts65Dn mice that have reported minimal, null, or negative effects of pure EGCG administration on cognitive outcomes (Stringer et al. 2015, 2017). The genetic differences between mouse models may underlie these discrepant results. Looking across studies, the administration of Mega Green Tea Extract (MGTE) Lightly Caffeinated (45% EGCG) improved MWM and NOR deficits in both Ts65Dn and TgDyrk1a mice (de la Torre et al. 2014). This treatment reduced *Dyrk1a* kinase activity in TgDyrk1a animals, suggesting that *Dyrk1a* overexpression and subsequent increased kinase activity could be driving these behavioral deficits in the transgenic mice. However, the same study did not report the effect of this treatment on *Dyrk1a* kinase activity in Ts65Dn mice. Thus, from this study, it remains unknown whether *Dyrk1a* activity is correlated with Ts65Dn behavioral deficits or improvement from treatment with EGCG‐containing supplements.

## Pure EGCG Administration has Failed to Reduce Dyrk1a‐related Kinase Activity In Vivo

There are only a few studies that have administered EGCG in vivo and reported effects on Dyrk1a kinase levels [Table 3]. A crucial gap in current research is that a decrease in Dyrk1a kinase activity in Ts65Dn mice (with more than *Dyrk1a* at dosage imbalance) has not been specifically attributed to EGCG. Reduced Dyrk1a kinase activity was shown in the hippocampus of young adult transgenic TgDyrk1a male and female mice following administration of an EGCG‐containing supplement (Mega Green Tea Extract‐ 45% EGCG, 98% polyphenols) (Pons‐Espinal et al. 2013; de la Torre et al. 2014). However, until experimental studies in trisomic mouse models demonstrate the extent, duration, and dose‐dependency of inhibition of Dyrk1a by EGCG in specific brain regions (or other tissues) that is directly linked to improved phenotypes, there will be uncertainty as to whether EGCG inhibition of Dyrk1a can account mechanistically for any therapeutic outcomes. Future studies need to identify when and where Dyrk1a overexpression occurs in trisomic mice, and show that EGCG normalizes Dyrk1a kinase activity at relevant times in relevant brain regions. Some contravening in vivo data in mice challenge the hypothesis that EGCG inhibits brain Dyrk1a activity to improve functional outcomes. For example, using a radioactive‐based assay, no significant differences in Dyrk1a‐related kinase activity levels were seen between euploid and Ts65Dn mice at 6 weeks of age, nor did EGCG treatment have an effect on these levels (Stringer et al. 2015). Several studies have reported methodological variations of Dyrk1a kinase assays (de la Torre et al. 2014; Stringer et al. 2015, 2017), and it will be important to further develop these assays in trisomic mouse models, as well as determining their specificity. In addition, further exploration into potential biomarkers, such as the plasma biomarker homocysteine (Hcy), should be examined as a potential measurement of treatment efficacy (Noll et al. 2009; de la Torre et al. 2014). Increased Dyrk1a protein levels in the liver of Ts65Dn mice have been correlated with decreased levels of plasma Hcy (Noll et al. 2009). In addition, there is a significant correlation between increased levels of Dyrk1a protein in the brain, and decreased levels of Hcy. Although 1 month of treatment with an EGCG‐containing supplement normalized plasma Hcy levels (de la Torre et al. 2014), it is unsure if these changes in Hcy levels are directly linked to changes in Dyrk1a activity. Future studies should determine whether alternate Dyrk1a inhibitors affect Hcy levels, in addition to determining if age of the mice affects the relationship between Dyrk1a protein levels and Hcy levels.

## There is not a Consistent Dose/Route of EGCG Administration to Inhibit Dyrk1a Activity

The dose and route of EGCG administration is also highly variable across studies. Calculations of the amount of EGCG administered in any dosing regimen must account for the differences in the amount of EGCG in the various EGCG‐containing supplements used, and should also determine the amount of the other catechins included in those supplements. The route of administration can also critically determine the amount of EGCG delivered to the tissue. When studies differ in the routes of administration, the temporal profile of EGCG levels reached in various tissue compartments and the within‐day variation in those levels is a key issue. The amount of EGCG delivered to the subject may either be directly controlled either by the experimenter (e.g., gavage or injection) or by the subject (oral consumption in the drinking water). Many studies administer EGCG via the drinking water, and the average intake is usually recorded over days to determine the average EGCG consumption. When EGCG delivery is controlled by the daily pattern of fluid consumption of the mouse, the levels of EGCG in the blood and tissues are difficult to establish or monitor, and will vary dramatically over the day by factors inherent to the subjects, including circadian regulation of drinking and pharmacokinetic differences in EGCG absorption, distribution, and metabolism. With this route of administration, EGCG levels in the tissues, if they do reach measurable levels, will vary substantially over time, but this source of variation is generally not accounted for or monitored when behavioral or biological endpoints are obtained. Another source of uncertainty about the dosage of EGCG is its instability in solution. EGCG undergoes rapid degradation in water, and solutions made from 95% EGCG lose approximately 80% of their initial concentration after just 48 hours. Acidifying the water by addition of phosphoric acid (approximately 100 *μ*L/100 mL tap water) to EGCG solutions has been shown to stabilize EGCG and limit degradation to approximately 50% after 48 hours. This acidification has no effect on fluid consumption in the mice, thereby effectively increasing the daily amount of EGCG delivered via the drinking water (Stringer et al. 2015). All studies involving EGCG administration via the drinking water, whether of EGCG alone or of EGCG‐containing supplements, must account for degradation when calculating the daily dose of EGCG that animals receive.

The pharmacokinetics of EGCG need to be considered when deciding on a dose or route of administration. EGCG displays poor bioavailability, and is rapidly metabolized in the liver via methylation, glucuronidation, and sulfation (Lambert et al. 2003; Lu et al. 2003a,b;). EGCG bioavailability has been increased when it is encapsulated, or when the reactive hydroxyl groups are protected via the addition of a peracetate group (Landis‐Piwowar et al. 2007; Wu et al. 2013; Li et al. 2014). If the bioavailability of EGCG can be improved, measuring the levels of EGCG metabolites could be a useful biomarker for EGCG consumption. The poor bioavailability of EGCG in humans, however, is a significant obstacle for clinical application (Nakagawa and Miyazawa 1997; Lin et al. 2007). If large dosing was required to reach clinically relevant levels, it is uncertain if high levels of EGCG would lead to increased EGCG levels in the brain. While few adverse side effects have been reported during chronic EGCG administration, it is unknown if a build‐up of EGCG in the brain (if present) would lead to changes within the brain, or changes in a specific phenotype.

## An Optimal Age for EGCG Administration to Inhibit Dyrk1a Activity has not been Defined

A key question pertaining to treatment timing and length is determining whether a given EGCG treatment will affect the long‐term trajectory of various phenotypes. For example, if an EGCG treatment that is limited to a particular prenatal period in which trisomic Dyrk1a is overexpressed and subsequently rescues phenotypes in adolescence, this would suggest that the normalization of excessive Dyrk1a activity during a critical early period is capable of changing the full trajectory of the trisomic phenotypes. This raises the question of whether interventions early in development can yield enduring improvement of phenotypes of DS, potentially rescuing brain development in a manner that persists into adulthood. To date, only a few studies have administered EGCG pre or perinatally (Guedj et al. 2009; McElyea et al. 2016a). For example, EGCG treatment at gestational (G)7‐G8 improved craniofacial precursor phenotypes in E9.5 embryos, and improved cranial vault structure in these same mice at 6 weeks of age (McElyea et al. 2016a). A short‐term treatment with EGCG (25 mg/kg) from P3–P15 improved hippocampal neurogenesis at P15 (Stagni et al. 2016). However, when the animals were evaluated 1 month after the cessation of treatment, there were no improvements in either hippocampal neurogenesis or performance on the MWM (Stagni et al. 2016). This suggests that earlier treatment, a second postnatal therapeutic window, or a continuous treatment may be necessary.

While there is growing interest and support to administer therapies for DS at earlier stages of development (Stagni et al. 2015), the majority of studies discussed administer EGCG postnatally. This is primarily due to the many unknowns of when Dyrk1a protein expression levels are elevated in trisomic mice. In addition, there is a lack of knowledge about the effects of EGCG administration on the mother or fetus during gestational development. However, recent studies have started to isolate and identify the interplay between these two crucial components of information, in order to develop a rational basis for the choice of timing and duration of EGCG administration to trisomic mice. For instance, *Dyrk1a* mRNA expression is significantly increased at E9.5 in the first pharyngeal arch (PA1) and neural tube of Ts65Dn mice, yet was significantly decreased at E10 in the PA1 of Ts65Dn mice (Solzak et al. 2013; McElyea et al. 2016a). This temporally and spatially specific expression of *Dyrk1a* mRNA levels led to the development of a prenatal administration of EGCG covering the key period of increased expression of Dyrk1a to improve the craniofacial abnormalities evident in developing Ts65Dn embryos. Oral gavage of 200 mg/kg EGCG to pregnant dams twice daily during gestational days 7–8 improved the PA1 volume, and number of neural crest cells in E9.5 Ts65Dn embryos (McElyea et al. 2016a). While levels of *Dyrk1a* mRNA were not measured at E7–E8, these findings illustrate the value of administering treatment based on gene and/or protein expression during a specific developmental period in a trisomic mouse. Interestingly, McElyea et al. 2016a also administered a lower dose of EGCG through drinking tubes to pregnant Ts65Dn mice (~12 mg/kg/day), beginning at early gestation through E9.5. In stark contrast to the 200 mg/kg/day two‐day treatment, treatment via the drinking water over the 9.5 days post conception, delivering an average daily dose of EGCG of 12 mg/kg/day, did not improve craniofacial deficits in E9.5 Ts65Dn mice.

Strategies for developing postnatal EGCG treatment also need to place primary consideration on factors such as the dose, route, timing and durations of EGCG treatment, and these choices need to be guided by the tissue‐specific patterns of developmental expression and Dyrk1a activity. Seemingly small differences in EGCG treatment approaches can yield discrepant results that are difficult to reconcile. For example, EGCG treatment via the drinking water (~9 mg/kg/day) for 3 weeks beginning at P24 rescued Ts65Dn skeletal deficits, yet, drinking higher concentrations of EGCG (~50 mg/kg/day) for 7 weeks beginning at P24 did not rescue any skeletal or cognitive deficits (Blazek et al. 2015; Stringer et al. 2017). Furthermore, this dose and length of EGCG treatment significantly worsened bone tissue and structural measures in both Ts65Dn and euploid mice, including decreased cortical and mechanical (strength) parameters associated with bone. The discrepancies in outcome measures point to the need to develop rational strategies for therapies targeting Dyrk1a activity, and these new studies need to be guided first by knowing the tissue‐specific patterns of developmental expression and Dyrk1a activity, then providing independent confirmation that the doses of EGCG administered reach the tissues and inhibit Dyrk1a activity.

## Conclusions

The interest in developing therapeutics to improve or correct the deficits caused by Ts21 has exploded over the past decade. Even within the past few years, there have been advanced clinical trials, reports in the lay press of mothers self‐administering supplements during pregnancy, as well as crowd‐funded clinical studies examining prenatal pharmaceutical treatment for mothers who are pregnant with a child with DS (Baggot and Baggot 2014; Bacharach 2016). During these times of growing hope for therapeutic interventions for individuals with DS that may improve cognitive and intellectual outcomes, the responsibility of the research community is to provide evidence that is objective, verifiable, and replicable.

Two major gaps in knowledge need to be addressed to establish a mechanistic basis for treatments targeting excessive Dyrk1a activity using Dyrk1a inhibitors. First, the temporal and spatial expression of Dyrk1a protein and kinase activity in trisomic mouse models must be characterized in greater detail, and altered regulation of pathways of downstream targets of Dyrk1a needs to be identified in those tissues during periods of excessive Dyrk1a activity. A fundamental basis for this approach, then, is to identify when *Dyrk1a* is overexpressed in specific tissues at defined ages in trisomic mice, some of which may represent sensitive periods of developmental vulnerability that yield long‐lasting DS structural and functional phenotypes. This can then guide efforts to identify cellular and molecular signaling processes regulated by Dyrk1a that are disrupted by excessive Dyrk1a activity. A better understanding of the mechanisms by which *Dyrk1a* overexpression results in the neurodevelopmental deficits of DS would provide a rational basis for therapeutics based on targeted inhibition of Dyrk1a to produce enduring improvement of DS phenotypes. Second, the bioavailability, specificity, and dose‐dependent inhibition of Dyrk1a by candidate therapeutics (including EGCG) must be ascertained for specific tissues, and correlations between pharmacological actions and therapeutic outcomes need to be established. Prospects for Dyrk1a inhibition as a molecular therapy for DS will depend on advances in several key areas, including: 1) determining specific dosing regimens and routes of administration that produce dose‐dependent changes in drug concentrations in specific tissues that correlate with concentration‐dependent inhibition of Dyrk1a; and, 2) determining the extent to which therapeutic efficacy of treatment varies as a function of developmental timing and duration of treatment and the temporal profile of tissue‐specific inhibition of Dyrk1a. If EGCG or any other drug targeting trisomic *DYRK1A* is to be considered as a rational treatment for DS phenotypes, it is important that treatment efficacy be contingent on and optimized for inhibition of DYRK1A in brain (or other) tissues implicated in the deficits observed in DS. If confirmed in future research, molecular therapeutics centered around Dyrk1a inhibition may provide a means to improve the lives of individuals with DS.

## Supporting information


**Table S1.** Behavioral and developmental phenotypes of transgenic *Dyrk1a* mouse models.
**Table S2.** Gene expression of *Dyrk1a* in brain regions at various ages in mice.
**Table S3.** Anatomical and histological cerebellar phenotypes in Ts65Dn mice.
**Table S4.** Anatomical and histological hippocampal phenotypes in Ts65Dn mice.
