# Supplementary material for: Targeting trisomic treatments: optimizing Dyrk1a inhibition to improve Down syndrome deficits
Source: Mol Genet Genomic Med. 2017 Sep 20;5(5):451–65. doi: 10.1002/mgg3.334 (PMC5606891; doi:10.1002/mgg3.334)
Supplement: Supplementary file 1 — Table S1. Behavioral and developmental phenotypes of transgenic Dyrk1a mouse models. Table S2. Gene expression of Dyrk1a in brain regions at various ages in mice. Table S3. Anatomical and histological cerebellar phenotypes in Ts65Dn mice. Table S4. Anatomical and histological hippocampal phenotypes in Ts65Dn mice. [file MGG3-5-451-s001.pdf]

Mouse Model	Deficient Phenotype	Preserved Phenotype	Authors
BACTgDyrk1a	Increased brain weight (9W)<br>MWM Acquisition and probe (2M)<br>Increased LTP<br>Decreased LTD	Body weight (9W), Cued MWM, Rotarod training	(Ahn et al., 2006)
	Increased spine density of pyramidal PFC neurons (4-5M), Increased PSD95 protein levels in pyramidal PFC neurons (4-5M),<br>Decreased LTP,<br>Increased LTD	Spine length of pyramidal PFC Neurons (4-5M)	(Thomazeau et al., 2014)
	Rotarod (higher speeds), Accelerating Rod<br>Spontaneous Alternation in Y-maze<br>MWM latency, thigmotaxis, MWM probe	SHIRPA protocol, open field activity, rotarod at lower speeds, accelerating rod, cued MWM	(Souchet et al., 2014)
	Decreased body weight & length (P30)<br>Increased brain weight (weaning to adulthood)	Body weight (P15, P60)<br>Body length (P60)<br>Brain weight (P0)<br>Cerebellum size	(Guedj et al., 2012)
YACTgDyrk1a	MWM acquisition, reverse platform<br>Locomotor hypoactivity	LTP<br>PPI	(Smith et al., 1997)
	Passive avoidance acquisition (P15)<br>Locomotor hyperactivity<br>Increased brain weight (P21, adult)<br>Increased neuron cell size in the hippocampus and cortex (adult)<br>Increased sized of hypothalamus and septum	Locomotor activity thigmotaxis, rearing, grooming, jumping, sniffing<br>Brain weight (P3)<br>Body weight	(Branchi et al., 2004)
	Increased brain weight and volume (3M)<br>Increased thalamus-hypothalamus volume<br>Decreased NOR discrimination<br>Decreased BDNF protein levels in hippocampus	Spontaneous alternation in y-maze<br>Number of entries during y-maze task<br>NOR discrimination	(Guedj et al., 2009)
	Decreased protein levels TRKB<br>MWM acquisition, probe, MWM reversal (4M)<br>Fear conditioning	Cued MWM	(Chabert et al., 2004)
	Increased brain weight<br>Increased brain volume (P2, P7 through 6M)<br>Increased thalamus-hypothalamus volume (4.5W-23W)<br>Increased midbrain volume (14.5W)<br>Increased cortex volume (2-23W)<br>Increased hippocampal volume (9.3, 14.5, 23W)	Body weight<br>Brain volume (P7)<br>Midbrain volume (4.5, 9.3, 23W)<br>Hippocampal volume (2, 4.5W)	(Sebrié et al., 2008)
TgDyrk1a	Delayed Walking activity (P14, P21)<br>Delayed negative geotaxis (P7-P14)<br>Delayed Climbing ability (P7)<br>Wire suspension test (P7)	Weight/body length<br>Sensorimotor functions<br>Pivoting locomotion tes (P7, P10, P14, P21)<br>Walking activity (P7, P10)<br>Climbing activity (P10, P14)<br>Wire suspension test (P10, P14)	(Arque et al., 2013)
	Pivoting locomotion test (P10)<br>Walking activity (P7, P10)<br>Homing test (P14)<br>Wire suspension test (P14)<br>Coat hanger test (adult)	Brain weight<br>Sensorial & Reflexologic responses<br>Pivoting locomotion (P7, P14)<br>Walking activity (P14)	(Altafaj et al., 2001)

	Elevated Plus maze (adult)<br>MWM acquisition, probe, reversal (adult)<br><br>Hyperactive (~2, 4 & 6M)<br>Deficient on treadmill task (~2, 4 & 6M)<br>Increased PPI (6-9M)<br><br>Touch escape task (12-14M)<br>Rotating rod task acquisition (12-14M)<br>Treadmill task (3M)<br>Irregular swimming pattern, thigmotaxic (14-16M)<br><br>Shorter dendrites in cortical pyramidal cells (2M)<br>Less dendritic complexity, reduced spine density in cortical pyramidal cells (2M)<br><br>MWM latency, thigmotaxic, probe (4M)<br>NOR discrimination<br><br>MWM acquisition, probe trial (7W)<br>Decreased dendritic length and complexity (11W*)<br>Cell cycle deficits, delayed neuronal differentiation, decreased cell proliferation rate (~7-11W)	PPI (~2-3M)<br><br>SHIRPA protocol (12-14M)<br>Gait pattern (12-14M)<br>Accelerating rotating rod test (12-14M)<br>Neuromuscular strength<br><br>Dendrite length (P7, P10)	(Ortiz-Abalia et al., 2008)<br><br>(de Lagrán et al., 2004)<br><br>(De Lagran et al., 2012)<br><br>(de la Torre et al., 2014)<br><br>(Pons-Espinal et al., 2013)
Dyrk1a +/-	Prehensile reflex (20-24M)<br>Coat-hanger test (20-24M)<br>Balance beam (20-24M)<br>Walking pattern (20-24M)<br>Accelerated rotarod test (4RPM) (20-24M)<br>Learning efficiency index (RAWM) (20-24M)<br><br>Decreased dendritic complexity, dendritic length, spine density in cortical pyramidal cells (7M)<br><br>Decreased body weight and length (birth into adulthood)<br>Decreased cerebellum weight<br>Decreased size of cerebral cortex, olfactory bulb, hypothalamus, pons, cerebellum<br><br>Pivoting (P7)<br>Climbing score (P14)<br>Paw print/Gait test<br>Decreased speed, acitivty in open field (5-7M)<br>Coat hanger task (5-7M)<br>Vertical grip strength (5-7M)<br><br>Decreased activity on wheel running (20-24M)	SHIRPA protocol (20-24M)<br>Acquisition roatarod test (20-24M)<br>Accelerated rotarod (7, 10, 12, 24, 34RPM) (20-24M)<br>Thickness CA3 (24M)<br>Volume CA3 (24M)<br><br>SHIRPA protocol<br><br>Pivoting (P10, P14)<br>Walking latency (P7, P10, P14)<br>Climbing score (P10)<br>Wire suspension test (P10, P14)<br>Stationary beam task (5-7M)<br>Accelerating rod task (5-7M)<br>Horizontal grip strength (5-7M)	(Arqué et al., 2008)<br><br>(Benavides-Piccione et al., 2005)<br><br>(Fotaki et al., 2002)<br><br>(Fotaki et al., 2004)<br><br>(Martinez de Lagran et al., 2007)

Supplemental Table 1: Behavioral and developmental phenotypes of transgenic *Dyrk1a* mouse models. Abbreviations: MWM (Morris Water Maze), M (Month), P (Postnatal Day), LTP (Long Term Potentiation), LTD (Long Term Depression), PFC (Prefrontal Cortex), PPI (Pre-Pulse Inhibition), PFC (Prefrontal Cortex), NOR (Novel Object Recognition)

Age	Sex	Type of Tissue	Level of Expression	Authors
P30	male and female	Cerebral Cortex	High	(Lizio et al., 2015)
Adult	male	Cerebral Cortex	High	(Lizio et al., 2015)
P30	male and female	cerebellum	High	(Lizio et al., 2015)
Adult	male	Cerebellum	Low	(Lizio et al., 2015)
P77	male	Brain	High	(Soumillon et al., 2013)
PD75	male and female	Brain	High	(Barbosa-Morais et al., 2012)
Adult	male	Dentate Gyrus	Low	(Ramos et al., 2013)
P0	male and female	Hippocampus	High	(Lizio et al., 2015)
Adult		Hippocampus	Medium	(Keane et al., 2011)
Adult	male	Hippocampus	Low	(Lizio et al., 2015)

Supplemental Table 2: Gene expression of *Dyrk1a* in brain regions at various ages in mice. Data was compiled from the EMBL-EBI Expression Atlas

	P0	P2	P6	P14-adulthood	P30	P45	P112	P120 - onward
**Deficient Phenotype**	reduced number of mitotic GCP (3)	reduced cerebellum volume (9)	Smaller cerebellar size (3)	IGL thinner (3)	Fewer labeled cells in the IGL (9)	reduced cerebellar volume (5)	Decreased cerebellar area, GC density (11)	Reduced cerebellar volume (10)
		EGL volume and total number of GCP in the EGL were smaller; fewer BrdU labeled cells (9)	GC density in IGL is reduced (3)		Smaller percentage of BrdU and NeuN cells (9)	Increased GC surface area (12)		Reduced density of GC's and PC's (10)
		Higher rates of apoptosis (based on appearance) (9)	Reduced number of mitotic figures in EGL (3)					Decreased IGL, ML area (10)
		Longer cell cycle; specifically at G1 and G2 phase (9)	Reduced GCP and mitotic cells (3)					
		Reduced thickness of the external and interal granular layers, cell denisty in the exteranl granular, purkinje cell and internal granular layers; cerebellar lobes had shallower fissures (5)						
**Preserved Phenotype**	Cerebellum same size (3)	No differences in S or M phase length (9)	No differences in apoptosis (3)					
	Number of GCP (3)							
	Normal B-galactosidase activity (3)							

Supplemental Table 3: Anatomical and histological cerebellar phenotypes in Ts65Dn mice. Abbreviations: GCP (Granule Cell Precursors), IGL (Internal Granular Layer), EGL (External Granule Layer), ML (Molecular Layer)
Sources: 1) (Najas et al., 2015); 2) (Chakrabarti et al., 2007); 3) (Roper et al., 2006); 4) (Contestabile et al., 2007); 5) (Guidi et al., 2014); 6) (Lorenzi and Reeves, 2006); 7) (Bianchi et al., 2010); 8) (Clark et al., 2006); 9) (Contestabile et al., 2009); 10) (Baxter et al., 2000); 11) (Das et al., 2013); 12) (Usowicz and Garden, 2012)

	E13.5-E18.5	P2	P6	P8	P15	P18	P30	P45	2-5 Months	5 months+
**Deficient Phenotype**		fewer cells in granule cell layer, hillus and molecular layer (4)	Fewer granule cells in DG; lower granule cell density; fewer mitotic cells (6)	decreased levels of SY38 and PSD95 protein in hippocampus and cortex (2)	proliferation in the SGZ and SVZ (7)	Ts6Dn had fewer cells in the hillus (2)	Fewer cells only in the granule cell layer (2)	Reduced Volume; Fewer Ki67 cells in SVZ & SGZ (7)	Ts65Dn showed proliferation deficits (8)	Mean neuron number was decreased in DG, higher in CA3 (14)
	in CA3, longer cell cycle (2)	volume of granule cell layer was smaller in Ts65Dn mice (4)		fewer PSD95 synapses in CA3, no differences in SY38 (2)	Fewer apoptotic cells in the DG, and a similar number in the SVZ (measured using cells that express caspase-3) (7)		More cells with an astrocytic phenotype (4)	higher levels of P45, reduced levels of phosphorylated Erk1/2 (5)	Ts65Dn mice showed fewer clusters of cells (8)	12-13month old Ts65Dn presented reduced DG volume, SGZ area, number of granule neurons, and number of BrdU positive cells (16)
	reduced hippocampal thickness (2)	Ts65Dn mice had larger percent number of cells in G2 phase, smaller percent in M phase (4)		fewer PSD95 & SY38 synapses in CA1 (2)	Granule cell layer had a smaller volume, reduced granule cell density and reduced number of granule cells (7)		Smaller number of granule cells & GC density (4)	Fewer BrdU cells, NeuN/BrdU labeled cells, astroctyes, reduced cell density, cell survival deficits (7)	fewer granule cells in DG; reduced volume of GCL (8)	16-23month old Ts65Dn presented decreased neuronal density in CA1, total synapse density in DG (17)
		reduced volume of granule cell layer, reduced granule cell density and reduced number of granule cells; reduced volume of CA3 & CA1, reduced pyramidal cell density and reduced number of pyramidal neurons (5)			lower expression of 5-Ht1A receptor in hippocampus (7)		Decreased BrdU and NeUN labeled cells in DG (22)	Smaller granule cell layer volume, reduced granule cell density, reduced granule cell number in Ts mcie (7)	Fewer GC's in DG (11)	13-17month old Ts65Dn presented fewer DCX positive cells (18)
		Ts mice have reduced expression of 5-HT1A-R in DG and SVZ (5)			BDNF expression is lower in hippocampus of Ts mice, as well as NGF expression (7)			Dendritic trees with reduced length and fewer branches (5)	Reduced LTP, increased LTD (13)	13-15month old Ts65Dn present reduced cell proliferation and density of surviving cells (21)
		Ts mice have reduced proliferation in DG (5)			Higher 5HT metabolite concentration (7)			Reduced protein levels of SYN and PSD95; also reduction in immunoreactivity (5)	Deficits in LTP (15,20)	
								Decreased BrdU and NeUN labeled cells in DG (22)		
**Preserved Phenotype**		no differences in apoptotic cell death (4)	no differences in GCL or PCL; pyramidal cells or density of pyramidal cells; no differences in apoptosis in DG; similar mitotic index (6)		5-HT concentration (7)	# of cells in granule cell layer (4)	No differences in cells with neuronal phenotype (4)		no differences in number of pyramidal cells in PCL; no volume differences in PCL; or granule and pyramidal cell densities; no differences in number of pyramidal cells in either CA1 or CA3; similar volumes and neuronal densities in CA1, CA3 and DG (6)	Hippocampal volumes no different than controls (except for CA2) (14)
						no differences in apoptotic cell death (4)	no difference in volume of granule cell layer (4)		No differences in HFS-induced LTP (15)	At 2-3, 6, 12, 16 and 21 months, no differences in brain weight (19)

						At P22, no differences in brain weight (19)			No differences in number of proliferating or surviving cells (21)	

Supplemental Table 4. Anatomical and histological hippocampal phenotypes in Ts65Dn mice. Abbreviations: GCL (Granule Cell Layer), PCL (Pyramidal Cell Layer), GC (Granule Cell), SVZ (Subventricular Zone), DG (Dentate Gyrus). Sources: 1) (Najas et al., 2015); 2) (Chakrabarti et al., 2007); 3) (Roper et al., 2006); 4) (Contestabile et al., 2007); 5) (Guidi et al., 2014); 6) (Lorenzi and Reeves, 2006); 7) (Bianchi et al., 2010); 8) (Clark et al., 2006); 9) (Contestabile et al., 2009); 10) (Baxter et al., 2000); 11) (Das et al., 2013); 12) (Usowicz and Garden, 2012); 13)(Siarey et al., 1999); 14) (Insausti et al., 1998); 15) (Costa and Grybko, 2005); 16) (Llorens-Martin et al., 2010); 17) (Kurt et al., 2004); 18) (Velazquez et al., 2013); 19) (Belichenko et al., 2004); 20) (Kleschevnikov et al., 2004); 21) (Rueda et al., 2005); 22) (Chakrabarti et al., 2011)

References

Ahn, K.-J., Jeong, H.K., Choi, H.-S., Ryoo, S.-R., Kim, Y.J., Goo, J.-S., Choi, S.-Y., Han, J.-S., Ha, I., Song, W.-J., 2006. DYRK1A BAC transgenic mice show altered synaptic plasticity with learning and memory defects. Neurobiology of disease 22, 463-472.

Altafaj, X., Dierssen, M., Baamonde, C., Martí, E., Visa, J., Guimerà, J., Oset, M., González, J.R., Flórez, J., Fillat, C., 2001. Neurodevelopmental delay, motor abnormalities and cognitive deficits in transgenic mice overexpressing Dyrk1A (minibrain), a murine model of Down’s syndrome. Human Molecular Genetics 10, 1915-1923.

Arque, G., Casanovas, A., Dierssen, M., 2013. Dyrk1A is dynamically expressed on subsets of motor neurons and in the neuromuscular junction: possible role in Down syndrome. PloS one 8, e54285.

Arqué, G., Fotaki, V., Fernández, D., De Lagrán, M.M., Arbonés, M.L., Dierssen, M., 2008. Impaired spatial learning strategies and novel object recognition in mice haploinsufficient for the dual specificity tyrosine-regulated kinase-1A (Dyrk1A). PLoS One 3, e2575.

Barbosa-Morais, N.L., Irimia, M., Pan, Q., Xiong, H.Y., Gueroussov, S., Lee, L.J., Slobodeniuc, V., Kutter, C., Watt, S., Çolak, R., 2012. The evolutionary landscape of alternative splicing in vertebrate species. Science (New York, N.Y.) 338, 1587-1593.

Baxter, L.L., Moran, T.H., Richtsmeier, J.T., Troncoso, J., Reeves, R.H., 2000. Discovery and genetic localization of Down syndrome cerebellar phenotypes using the Ts65Dn mouse. Human Molecular Genetics 9, 195-202.

Belichenko, P.V., Masliah, E., Kleschevnikov, A.M., Villar, A.J., Epstein, C.J., Salehi, A., Mobley, W.C., 2004. Synaptic structural abnormalities in the Ts65Dn mouse model of Down Syndrome. Journal of Comparative Neurology 480, 281-298.

Benavides-Piccione, R., Dierssen, M., Ballesteros-Yanez, I., de Lagran, M.M., Arbones, M., Fotaki, V., DeFelipe, J., Elston, G., 2005. Alterations in the phenotype of neocortical pyramidal cells in the Dyrk1A+/− mouse. Neurobiology of disease 20, 115-122.

Bianchi, P., Ciani, E., Guidi, S., Trazzi, S., Felice, D., Grossi, G., Fernandez, M., Giuliani, A., Calza, L., Bartesaghi, R., 2010. Early pharmacotherapy restores neurogenesis and cognitive performance in the Ts65Dn mouse model for Down syndrome. Journal of Neuroscience 30, 8769-8779.

Branchi, I., Bichler, Z., Minghetti, L., Delabar, J.M., Malchiodi-Albedi, F., Gonzalez, M.-C., Chettouh, Z., Nicolini, A., Chabert, C., Smith, D.J., 2004. Transgenic mouse in vivo library of human Down syndrome critical region 1: association between DYRK1A overexpression, brain development abnormalities, and cell cycle protein alteration. Journal of Neuropathology & Experimental Neurology 63, 429-440.

Chabert, C., Jamon, M., Cherfouh, A., Duquenne, V., Smith, D.J., Rubin, E., Roubertoux, P.L., 2004. Functional analysis of genes implicated in Down syndrome: 1. Cognitive abilities in mice transpolygenic for Down Syndrome Chromosomal Region-1 (DCR-1). Behavior genetics 34, 559-569.

Chakrabarti, L., Galdzicki, Z., Haydar, T.F., 2007. Defects in embryonic neurogenesis and initial synapse formation in the forebrain of the Ts65Dn mouse model of Down syndrome. The Journal of neuroscience 27, 11483-11495.

Chakrabarti, L., Scafidi, J., Gallo, V., Haydar, T.F., 2011. Environmental enrichment rescues postnatal neurogenesis defect in the male and female Ts65Dn mouse model of Down syndrome. Developmental neuroscience 33, 428-441.

Clark, S., Schwalbe, J., Stasko, M.R., Yarowsky, P.J., Costa, A.C., 2006. Fluoxetine rescues deficient neurogenesis in hippocampus of the Ts65Dn mouse model for Down syndrome. Experimental neurology 200, 256-261.

Contestabile, A., Fila, T., Bartesaghi, R., Ciani, E., 2009. Cell cycle elongation impairs proliferation of cerebellar granule cell precursors in the Ts65Dn mouse, an animal model for Down syndrome. Brain Pathology 19, 224-237.

Contestabile, A., Fila, T., Ceccarelli, C., Bonasoni, P., Bonapace, L., Santini, D., Bartesaghi, R., Ciani, E., 2007. Cell cycle alteration and decreased cell proliferation in the hippocampal dentate gyrus and in the neocortical germinal matrix of fetuses with Down syndrome and in Ts65Dn mice. Hippocampus 17, 665-678.

Costa, A.C., Grybko, M.J., 2005. Deficits in hippocampal CA1 LTP induced by TBS but not HFS in the Ts65Dn mouse: a model of Down syndrome. Neuroscience letters 382, 317-322.

Das, I., Park, J.-M., Shin, J.H., Jeon, S.K., Lorenzi, H., Linden, D.J., Worley, P.F., Reeves, R.H., 2013. Hedgehog agonist therapy corrects structural and cognitive deficits in a Down syndrome mouse model. Science Translational Medicine 5, 201ra120-201ra120.

de la Torre, R., Sola, S., Pons, M., Duchon, A., Lagran, M.M., Farré, M., Fitó, M., Benejam, B., Langohr, K., Rodriguez, J., 2014. Epigallocatechin-3-gallate, a DYRK1A inhibitor, rescues cognitive deficits in Down syndrome mouse models and in humans. Molecular nutrition & food research 58, 278-288.

de Lagrán, M.M., Altafaj, X., Gallego, X., Martı, E., Estivill, X., Sahun, I., Fillat, C., Dierssen, M., 2004. Motor phenotypic alterations in TgDyrk1a transgenic mice implicate DYRK1A in Down syndrome motor dysfunction. Neurobiology of disease 15, 132-142.

De Lagran, M.M., Benavides-Piccione, R., Ballesteros-Yañez, I., Calvo, M., Morales, M., Fillat, C., DeFelipe, J., Ramakers, G., Dierssen, M., 2012. Dyrk1A influences neuronal morphogenesis through regulation of cytoskeletal dynamics in mammalian cortical neurons. Cerebral cortex, bhr362.

Fotaki, V., Dierssen, M., Alcántara, S., Martínez, S., Martí, E., Casas, C., Visa, J., Soriano, E., Estivill, X., Arbonés, M.L., 2002. Dyrk1A haploinsufficiency affects viability and causes developmental delay and abnormal brain morphology in mice. Molecular and Cellular Biology 22, 6636-6647.

Fotaki, V., Martinez De Lagran, M., Estivill, X., Arbones, M., Dierssen, M., 2004. Haploinsufficiency of Dyrk1A in mice leads to specific alterations in the development and regulation of motor activity. Behavioral neuroscience 118, 815.

Guedj, F., Pereira, P.L., Najas, S., Barallobre, M.-J., Chabert, C., Souchet, B., Sebrie, C., Verney, C., Herault, Y., Arbones, M., 2012. DYRK1A: a master regulatory protein controlling brain growth. Neurobiology of disease 46, 190-203.

Guedj, F., Sébrié, C., Rivals, I., Ledru, A., Paly, E., Bizot, J.C., Smith, D., Rubin, E., Gillet, B., Arbones, M., 2009. Green tea polyphenols rescue of brain defects induced by overexpression of DYRK1A. PLoS One 4, e4606.

Guidi, S., Stagni, F., Bianchi, P., Ciani, E., Giacomini, A., De Franceschi, M., Moldrich, R., Kurniawan, N., Mardon, K., Giuliani, A., Calza, L., Bartesaghi, R., 2014. Prenatal pharmacotherapy rescues brain development in a Down's syndrome mouse model. Brain : a journal of neurology 137, 380-401.

Insausti, A., Megıas, M., Crespo, D., Cruz-Orive, L., Dierssen, M., Vallina, T., Insausti, R., Florez, J., 1998. Hippocampal volume and neuronal number in Ts65Dn mice: a murine model of Down syndrome. Neuroscience letters 253, 175-178.

Keane, T.M., Goodstadt, L., Danecek, P., White, M.A., Wong, K., Yalcin, B., Heger, A., Agam, A., Slater, G., Goodson, M., 2011. Mouse genomic variation and its effect on phenotypes and gene regulation. Nature 477, 289-294.

Kleschevnikov, A.M., Belichenko, P.V., Villar, A.J., Epstein, C.J., Malenka, R.C., Mobley, W.C., 2004. Hippocampal long-term potentiation suppressed by increased inhibition in the Ts65Dn mouse, a genetic model of Down syndrome. The Journal of neuroscience 24, 8153-8160.

Kurt, M.A., Kafa, M.I., Dierssen, M., Davies, D.C., 2004. Deficits of neuronal density in CA1 and synaptic density in the dentate gyrus, CA3 and CA1, in a mouse model of Down syndrome. Brain research 1022, 101-109.

Lizio, M., Harshbarger, J., Shimoji, H., Severin, J., Kasukawa, T., Sahin, S., Abugessaisa, I., Fukuda, S., Hori, F., Ishikawa-Kato, S., 2015. Gateways to the FANTOM5 promoter level mammalian expression atlas. Genome biology 16, 22.

Llorens-Martin, M., Rueda, N., Tejeda, G.S., Florez, J., Trejo, J.L., Martínez-Cué, C., 2010. Effects of voluntary physical exercise on adult hippocampal neurogenesis and behavior of Ts65Dn mice, a model of Down syndrome. Neuroscience 171, 1228-1240.

Lorenzi, H.A., Reeves, R.H., 2006. Hippocampal hypocellularity in the Ts65Dn mouse originates early in development. Brain research 1104, 153-159.

Martinez de Lagran, M., Bortolozzi, A., Millan, O., Gispert, J.D., Gonzalez, J.R., Arbones, M.L., Artigas, F., Dierssen, M., 2007. Dopaminergic deficiency in mice with reduced levels of the dual-specificity tyrosine-phosphorylated and regulated kinase 1A, Dyrk1A+/−. Genes, Brain and Behavior 6, 569-578.

Najas, S., Arranz, J., Lochhead, P.A., Ashford, A.L., Oxley, D., Delabar, J.M., Cook, S.J., Barallobre, M.J., Arbonés, M.L., 2015. DYRK1A-mediated cyclin D1 degradation in neural stem cells contributes to the neurogenic cortical defects in down syndrome. EBioMedicine 2, 120-134.

Ortiz-Abalia, J., Sahún, I., Altafaj, X., Andreu, N., Estivill, X., Dierssen, M., Fillat, C., 2008. Targeting Dyrk1A with AAVshRNA attenuates motor alterations in TgDyrk1A, a mouse model of Down syndrome. The American Journal of Human Genetics 83, 479-488.

Pons-Espinal, M., Martinez de Lagran, M., Dierssen, M., 2013. Environmental enrichment rescues DYRK1A activity and hippocampal adult neurogenesis in TgDyrk1A. Neurobiology of Disease 60, 18-31.

Ramos, A.D., Diaz, A., Nellore, A., Delgado, R.N., Park, K.-Y., Gonzales-Roybal, G., Oldham, M.C., Song, J.S., Lim, D.A., 2013. Integration of genome-wide approaches identifies lncRNAs of adult neural stem cells and their progeny in vivo. Cell stem cell 12, 616-628.

Roper, R.J., Baxter, L.L., Saran, N.G., Klinedinst, D.K., Beachy, P.A., Reeves, R.H., 2006. Defective cerebellar response to mitogenic Hedgehog signaling in Down's syndrome mice. Proceedings of the National Academy of Sciences of the United States of America 103, 1452-1456.

Rueda, N., Mostany, R., Pazos, Á., Flórez, J., Martínez-Cué, C., 2005. Cell proliferation is reduced in the dentate gyrus of aged but not young Ts65Dn mice, a model of Down syndrome. Neuroscience letters 380, 197-201.

Sebrié, C., Chabert, C., Ledru, A., Guedj, F., Po, C., Smith, D.J., Rubin, E., Rivals, I., Beloeil, J.C., Gillet, B., 2008. Increased dosage of DYRK1A and brain volumetric alterations in a YAC model of partial trisomy 21. The anatomical record 291, 254-262.

Siarey, R.J., Carlson, E.J., Epstein, C.J., Balbo, A., Rapoport, S.I., Galdzicki, Z., 1999. Increased synaptic depression in the Ts65Dn mouse, a model for mental retardation in Down syndrome. Neuropharmacology 38, 1917-1920.

Smith, D.J., Stevens, M.E., Sudanagunta, S.P., Bronson, R.T., Makhinson, M., Watabe, A.M., O'Dell, T.J., Fung, J., Weier, H.U., Cheng, J.F., Rubin, E.M., 1997. Functional screening of 2 Mb of human chromosome 21q22.2 in transgenic mice implicates minibrain in learning defects associated with Down syndrome. Nat Genet 16, 28-36.

Souchet, B., Guedj, F., Sahún, I., Duchon, A., Daubigney, F., Badel, A., Yanagawa, Y., Barallobre, M.J., Dierssen, M., Yu, E., 2014. Excitation/inhibition balance and learning are modified by Dyrk1a gene dosage. Neurobiology of disease 69, 65-75.

Soumillon, M., Necsulea, A., Weier, M., Brawand, D., Zhang, X., Gu, H., Barthès, P., Kokkinaki, M., Nef, S., Gnirke, A., 2013. Cellular source and mechanisms of high transcriptome complexity in the mammalian testis. Cell reports 3, 2179-2190.

Thomazeau, A., Lassalle, O., Iafrati, J., Souchet, B., Guedj, F., Janel, N., Chavis, P., Delabar, J., Manzoni, O.J., 2014. Prefrontal deficits in a murine model overexpressing the down syndrome candidate gene dyrk1a. The Journal of Neuroscience 34, 1138-1147.

Usowicz, M.M., Garden, C.L., 2012. Increased excitability and altered action potential waveform in cerebellar granule neurons of the Ts65Dn mouse model of Down syndrome. Brain research 1465, 10-17.

Velazquez, R., Ash, J.A., Powers, B.E., Kelley, C.M., Strawderman, M., Luscher, Z.I., Ginsberg, S.D., Mufson, E.J., Strupp, B.J., 2013. Maternal choline supplementation improves spatial learning and adult hippocampal neurogenesis in the Ts65Dn mouse model of Down syndrome. Neurobiology of disease 58, 92-101.
